# Toward better understanding and management of chemobrain: the potential utilities of the MemTrax memory test

**DOI:** 10.1186/s12905-024-03251-4

**Published:** 2024-07-17

**Authors:** Yun Ma, Wenying Chai, Deyong Bu, Xuemin Feng, J. Wesson Ashford, Limei He, Ying Zheng, Curtis B. Ashford, Feng Li, Jun Li, Yuan Dong, Shumo Li, Xianbo Zhou

**Affiliations:** 1 Department of Breast Surgery, The First Affiliated Hospital of Kunming Medical University, Kunming, Yunnan 650032 China; 2 Department of Geriatric General Surgery, The First Affiliated Hospital of Kunming Medical University, Kunming, Yunnan China; 3 School of Public Health, Kunming Medical University, Kunming, Yunnan China; 4 Department of Psychiatry & Behavioral Sciences, Stanford University, War Related Illness & Injury Study Center, VA Palo Alto Health Care System, 3801 Miranda Ave, Palo Alto, CA USA; 5 Department of Neurology, The First Affiliated Hospital of Kunming Medical University, Kunming, Yunnan China; 6 MemTrax, LLC, Redwood City, CA USA; 7 Kunming Escher Technology Co. Ltd, Kunming, Yunnan China; 8 Department of Radiology, The First Affiliated Hospital of Kunming Medical University, Kunming, Yunnan China; 9 Center for Alzheimer’s Research, Washington Institute of Clinical Research, Vienna, VA USA; 10 AstraNeura, Co., Ltd, Shanghai, China

**Keywords:** MemTrax memory test, Breast cancer, Chemobrain

## Abstract

**Objective:**

To study the effects of chemotherapy on cognitive function in breast cancer patients, and to investigate the relationship of MemTrax test of memory and related functions to the FACT-Cog functional self-assessment for the evaluation and management of chemobrain.

**Methods:**

In this prospective cohort study, clinical information of pathologically confirmed female breast cancer patients who decided to receive chemotherapy were collected in a questionnaire which was developed for this study and provided as a supplementary file. The FACT-Cog self-assessment and MemTrax test were administered before and after the chemotherapy treatments. Patients with chemobrain were identified using published criteria based on FACT-Cog scores, and MemTrax scores from chemobrain patients were analyzed.

**Results:**

Fifty-six patients participated in this study, of which 41 participants completed 4 or more cycles of chemotherapy and were included in the final analyses here. Using the reported high end of minimal clinical differences (10.6 points) of FACT-Cog before and after chemotherapy, 18 patients suffered from chemobrain in this study. In these 18 chemobrain patients, no cognitive impairments were detected by MemTrax, which paradoxically demonstrated an improvement in the normal cognitive range.

**Conclusion:**

The cognitive impairment induced by chemotherapy in breast cancer patients is detectable by the FACT-Cog in a Chinese cohort but is not detected by the MemTrax memory test. The fact that the more objective MemTrax could not detect the impairment could alleviate patients’ concerns which in turn would be beneficial for patients’ mental health.

**Supplementary Information:**

The online version contains supplementary material available at 10.1186/s12905-024-03251-4.

Female breast cancer has surpassed lung cancer to become the most commonly diagnosed cancer worldwide and is now ranked 4^th^ among the leading causes of tumor-related death [1]. In recent years, the early diagnosis, early treatment and adjuvant therapy of breast cancer have made great progress, and the prognosis of breast cancer patients has been greatly improved, with the 5-year overall-survival (OS) rate of early breast cancer patients as high as 89 ~ 100% [2, 3]. Chemotherapy has been used in the clinical treatment of solid tumors for more than 50 years. Cytotoxic regimens can kill tumor cells, inhibit tumor cell proliferation and induce tumor cell apoptosis, and significantly improve the OS and disease-free-survival (DFS) rate of patients. Even now, chemotherapy is still one of the main adjuvant therapies for breast cancer patients.

Although chemotherapy can prolong OS and DFS in breast cancer patients, it has significant toxic side effects. In addition to conventional side effects such as nausea, vomiting, alopecia, bone marrow suppression, the cognitive impairment caused by chemotherapy regimens, also known as chemobrain, is an important problem that many tumor patients have to face after chemotherapy, especially in breast cancer patients [4]. Chemobrain can last for many years after chemotherapy, resulting in adverse effects on the mental health and quality of life of breast cancer patients, with severe cases even unable to work and live normally [4–6]. Currently the cause of chemobrain is still unknown, objective assessment tools and effective treatments are lacking [7, 8]. Our study aims to understand the effects of conventional chemotherapy regimens on cognitive impairment of Chinese breast cancer patients, the effectiveness of different assessment tools to detect chemobrain and any beneficial effects the more objective MemTrax test may provide the patients.

## Materials and methods

### Study population

Participants were enrolled continuously between September 2019 and April 2021. All participants were recruited through in-person discussion with patients who volunteered to participate in the study on the in-patient ward before their first chemotherapy session in the First Affiliated Hospital of Kunming Medical University. Inclusion criteria: participants who were pathological diagnosed and underwent breast cancer surgery; prescribed to receive conventional chemotherapy such as ECX4-T (H) X4 (Epirubicin and cyclophosphamide every 3 weeks for 4 cycles followed or preceded by docetaxel (trastuzumab) every 3 weeks for 4 cycles)and TCX4 (docetaxel and cyclophosphamide every 3 weeks for 4 cycles); female aged between 35-year-old to 70-year-old, without previous chemotherapy, endocrinotherapy or radiotherapy history and had the ability to understand and sign informed consent. Exclusion criteria: Patients with brain-metastasis or stage IV cancer, psychiatric-symptoms, dementia, or other conditions that are known to affect cognitive function. This study was performed according to the Helsinki declaration of 1975 and was approved by the ethical committee of the first Affiliated Hospital of Kunming Medical University in Kunming, Yunnan, China. All participants voluntarily signed an informed consent form.

#### The Functional Assessment of Cancer Therapy Cognitive Function (FACT-Cog) (Version 3, Simplified Chinese) and subscales

FACT-Cog is a subjective assessment instrument designed to evaluate the effects of cancer patients’ perceived cognitive deterioration in their health-related quality of life [7] which is owned and copyrighted by David Cella, Ph.D. who has kindly granted a license to use the Simplified Chinese version of the FACT-Cog (Version 3) which has been used in this not-for-profit study. English and Chinese versions of FACT-Cog have been validated within the Asian breast cancer population [8]. FACT-Cog is a 37-item questionnaire covering six cognitive domains: memory, concentration, mental acuity, verbal fluency, functional interference, and multitasking ability which is organized in 4 sections/subscales: Perceived Cognitive Impairments (CogPCI, score range 0–72. There are 2 items in this subscale that are not scored but included as internal consistent controls), Comments From Others (CogOth, score range 0–16), Perceived Cognitive Abilities (CogPCA, score range 0–28. There are 2 items in this subscale that are not scored but included as internal consistent controls) and Impact Of Perceived On Quality Of Life (CogQOL, score range 0–16). In the subscales CogPCI and CogOth, the participants report on a 5-point Likert scale ranging from 0, ‘‘never,’’ to 4, ‘‘several times a day,’’ the frequency of each occurrence over the 7 days preceding the test. In the subscales of CogPCA and CogQOL, the responses are rated on a 5-point severity scale ranging from 0, ‘‘not at all,’’ to 4, ‘‘very much.’’ The individual subscale scores are summed to determine the FACT-Cog total score, ranging from 0 to 132, with higher scores indicating better subjective cognitive functioning.

#### MemTrax test procedures and output metrics

Detailed description of the theory and design of MemTrax has been published previously [9]. Briefly, with each MemTrax test, a series of 50 images are shown: 25 new images and 25 repeated images. Each picture is presented on the screen for 3 seconds or until a behavioral response, at which time the next picture is shown immediately. The participants are instructed to respond by either pressing the space-bar when performing the test on a computer or touching the screen when a smart phone is used, but only when presented with a repeated picture and as quickly as possible. The program automatically calculates the total percent correct (MTx- %C) and the average response time (MTx-RT) as outcome measures. MemTrax can be administered on the Chinese social media platform WeChat using its mini-program version on a phone or web on a computer on a cloud server in China (http://www.memtrax.com.cn) [10] and MemTrax is also available at www.memtrax.org. All the participants in our study completed the MemTrax on WeChat on a phone.

### Data collection

A general questionnaire survey was administered to collect demographic and clinical information including age, educational level, previous medical history, medication history, hobbies, body mass index (BMI), sleep disorders, craniocerebral injury history and family history of dementia. Regarding the breast cancer, the pathological type, TNM stage and chemotherapy regimen were recorded.

Following completion of the questionnaire survey, the FACT-Cog and MemTrax tests were administered, scored and uploaded into Excel spreadsheets by the researcher who administered the tests. Entries were verified by a colleague before the Excel files were saved for analyses.

The tests were administered one day before the first cycle of chemotherapy in person. The tests were completed before (in person) and one week (remotely via phone) after each additional cycle of chemotherapy until the completion of the chemotherapy cycles.

### Statistical analyses

There are at least two ways to determine the impact of changes in cognitive function: statistically or clinically meaningful differences [11]. While the norms for FACT-Cog in Chinese patient population are not established, the values are potentially useful for statistical comparison [11]. The minimal clinically important or meaningful difference for FACT-Cog has been reported in a study with 79.6% Chinese participants with reported values of ~ 6.9–10.6 points [11]corresponding to one third, half SD (standard deviation) and one SE. This study used the largest reported 10.6 points differential as criteria for chemobrain to ensure real impairment detection and performed statistical analyses on those patients who met this chemobrain criteria.

For normally distributed metric data, the “mean ± standard deviation” (x̄ ± s) was used, and the classified data were expressed by cases and percentages [n (%)]. Age and years of education were tested by t-test while the rest of the demographic information was tested with χ^2^. Paired t-test was used for the 18 cases of FACT-Cog cognitive function score and MTx-%C and MTx-RT before and after brain chemotherapy. The database was established by EpiData 3.1. The statistical analysis set was constructed after double entry. The data were analyzed by IBM SPSS 26.0. Statistically significant differences were set as *P* ≤ 0.05.

## Results

### Participants demographics

A total of 41 Chinese participants completed 4 or more cycles of chemotherapy and were included in the final analyses reported here. All participants were female, average age 47 ± 8 (range: 33–67) years. The average education was 12 ± 4 (range 3–21) years. 

The demographics for the 18 chemobrain and 23 cognitive normal (CN) after chemotherapy participants are shown in Table 1 where the age is found to be significantly older in the chemobrain participants (*P* < 0.04). The higher percentage of participants in the CN group who reported memory decline in the last 5 years did not reach statistical significance (*P* < 0.06).

**Table 1:** Demographics characteristics [*n*(%)/$\overline{x} \pm s$]

	CN (*n* = 23)	Chemobrain (*n* = 18)	*P*
Age	45.0 ± 6.8	50.0 ± 8.4	0.04
Year of education	12.0 ± 4.0	11.0 ± 3.0	0.50
Chemotherapy regimens			
ECX4-T (H)X4	11(52.4)	10(47.6)	0.87
TCX4	7(58.3)	5(41.7)	
ECX4	5(62.5)	3(37.5)	
Occupation			
White Collar	15(60.0)	10(40.0)	0.53
Blue Collar	8(50.0)	8(50.0)	
Living Condition			
Gregarious	22(55.0)	18(45.0)	0.56
Solitary	1(100.0)	0(0.0)	
Memory decline: last 2 years			
No	8(47.1)	9(52.9)	0.33
Yes	15(62.5)	9(37.5)	
Memory decline: last 5 years			
No	5(35.7)	9(64.3)	0.06
Yes	18(66.7)	9(33.3)	
History of anemia			
No	21(60.0)	14(40.0)	0.22
Yes	2(33.3)	4(66.7)	
Family history of dementia			
No	20(54.1)	17(45.9)	0.63
Yes	3(75)	1(25)	
Age	45 ± 7	50 ± 8	0.04
Year of education	12 ± 4	11 ± 3	0.5
Memory decline: last 2 years (Y/N)	15/8	9/9	0.3
Memory decline: last 5 years (Y/N)	18/5	9/9	0.06
Occupatio (White/Blue Collar)	15/8	10/8	0.5

*CN* Cognitive normal group

### FACT-Cog quantified chemobrain

Using the reported [11] high end of minimal clinically important differences (10.6 points) of FACT-Cog before and after chemotherapy, 18 patients out of 41 (44%) suffered from chemobrain, and the results of these participants’ before and after chemotherapy FACT-Cog total and sub-score are summarized in Table 2. FACT-Cog total, sub-scale COgPCI, CogOth, CogPCA and CogQoL scores are all significantly highly reduced with *p* < 0.001, < 0.001, = 0.045, < 0.001 and = 0.022, respectively in these 18 chemobrain patients. Comparing to the 23 patients who are cognitively normal after chemotherapy, the before score for the total FACT-Cog and all the subscores except for the CogQoL are statistically higher in the 18 chemobrain patients. After chemotherapy, the FACT-Cog total score, CogPCT and CogQoL subscores are significantly lower in the 18 than those of the 23 cognitive normal patients.

**Table 2:** FACT-Cog cognitive function scores before and after chemotherapy[$\overline{x} \pm s$]

	CN (*n* = 23)			Chemobrain (*n* = 18)				
	Before^*^	After^**^	*P*	Before^*^	After^**^	*P*	*P* ^*^	*P* ^**^
FACT-Cog	98 ± 17	101 ± 16	0.347	113 ± 13	89 ± 15	< 0.001	0.004	0.02
CogPCI	54 ± 11	56 ± 10	0.288	63 ± 7	50 ± 10	< 0.001	0.002	0.09
CogOth	15 ± 1	15 ± 1	0.257	16 ± 1	13 ± 5	0.05	0.03	0.1
CogPCA	19 ± 5	18 ± 6	0.438	22 ± 5	16 ± 5	< 0.001	0.05	0.2
CogQOL	11 ± 3	12 ± 3	0.365	12 ± 3	9 ± 3	0.002	0.124	0.03

*CN* Cognitive normal group, *CogPCI* Perceived cognitive impairments, *CogOth* Comments from others, *CogPCA* Perceived cognitive ability, *CogQOL* Impact of perceived cognitive impairments on quality of life, P*, *P* value comparing CN vs chemobrain groups’ prior to chemotherapy data, P**, *P* value comparing CN vs chemobrain groups’ after chemotherapy data

Multivariate regression analyses did not find that any of the data collected, including the demographic information, was significantly related to the chemobrain findings which these 18 participants suffered after chemotherapy (results not shown).

### Chemobrain is not reflected by the MemTrax memory test

The MemTrax scores in all 41 participants including the 18 chemobrain participants before and after chemotherapy are in the normal cognitive range of > 80% MTx-%C and < 1.4 s MTx-RT. There were no statistically differences detected between the two groups before chemotherapy. However, there were statistically significant differences in MTx-%C and MTx-RT, indicating small numerical improvement after chemotherapy in both groups as well as a statistical difference between the two groups after chemotherapy for reaction time where the 18 chemotherapy patients were significantly slower (*P* < 0.009) (Table 3).

**Table 3:** MemTrax Score in participants with and without chemobrain before and after chemotherapy[$\overline{x} \pm s$]

	CN (*n* = 23)			Chemobrain (*n* = 18)				
	Before^*^	After^**^	*P*	Before^*^	After^**^	*P*	*P* ^*^	*P* ^**^
MTx-%C(%)	90 ± 6	96 ± 4	< 0.001	89 ± 6	94 ± 5	< 0.001	0.8	0.3
MTx-RT(s)	1.17 ± 0.26	0.94 ± 0.14	< 0.001	1.18 ± 0.23	1.09 ± 0.21	0.004	0.9	0.009

*CN* Cognitive normal group, *MTx-%C* MemTrax percent correct, *MTx-RT* MemTrax mean reaction time, P* P value comparing CN vs chemobrain groups’ prior to chemotherapy data, P** P value comparing CN vs chemobrain groups’ after chemotherapy data

## Discussion

Chemobrain could be either a temporary or sustained damage to brain mechanisms underlying cognitive function of cancer patients induced by chemotherapy regimens [12], which is mainly manifested in the retardation of verbal memory, attention and reaction time, including memory loss, inattention and executive decline [13–16]. Cancer chemotherapy in rats is thought to cause cognitive impairment through adverse effects on the brain [17]. The incidence of chemobrain varies greatly in different studies, ranging from 16 to 80% [18–20] which is in line with the 44% in this study here. To our knowledge, this study is the first study of chemobrain in China. The exact etiology of chemobrain has not been clarified, and whether this condition is an objective phenomenon or subjective perception of patients has been controversial. In view of the different clinical manifestations and severity of chemobrain, how to accurately identify and quantify chemobrain is very important for clinicians and patients. Several cognitive assessment instruments such as Mini-Mental State Exam [21], Mini-Cog [22] and Montreal Cognitive Assessment (MoCA, [23]) fail to detect chemobrain, Dr. Wagner and her colleagues customized the FACT-Cog which proved to be able to detect chemobrain [7,24]. Dr. Cheung and her colleagues conducted the verification of the Chinese version of the FACT-Cog scale and the impact of chemotherapy on the cognitive function in a Chinese population in Singapore and found that the results were similar to those of studies in other cultures though some items are not culturally relevant to Chinese [8]. The FACT-Cog scales quantify the subjective impact of chemotherapy by asking patients to respond a series of 37 statements about their cognitive function, on a 5-point rating scale assessing “in the past 7 day” time frame (0 = Never; 1 = About once a week; 2 = Two to three times a week; 3 = Nearly every day; 4 = Several times a day). Objective, reliable and simple evaluation tools that could detect chemobrain would be a valuable contribution to research and clinical practice as well as the psychological well-being of affected patients. This study selected the ~ 2.5 min MemTrax memory test reported to be able to detect cognitive changes associated with aging [24, 25], and the test has been cross-validated with the MoCA for its ability to detect MCI due to AD, VaD and stroke [26–28]. MemTrax conforms to most of the parameters of the ideal digital cognitive testing system proposed by the International Working Group of MCI and AD experts [29].

In our study, the FACT-Cog was able to measure chemobrain in a Chinese female breast cancer cohort using the high end of the reported minimal clinically meaningful difference range of 6.9–10.6 points [11]. In contrast to the more subjective measures of FACT-Cog, MemTrax did not detect chemobrain in this study where the MemTrax scores before and after chemotherapy are all in the normal range in these same chemobrain patients as well as the cognitive normal patients. Interestingly, MemTrax outcome measures, MTx-%C and MTx-RT, demonstrated a statistically significant improvement after chemotherapy in all 41 participants though the improvement in reaction time in the chemobrain group is less than the cognitive normal group. The possible reasons for this apparent discrepancy between the results of the subjective FACT-Cog and the objective MemTrax measures could be that the cognitive function of breast cancer patients during chemotherapy is affected by emotional/psychological factors that are subjective which in-turn can only be detected by the subjective FACT-Cog but cannot be detected with a more objective instrument such as MemTrax. This finding is consistent with the inability of MMSE and MoCA to detect chemobrain [7]. The improvement of the normal range MemTrax performance after chemotherapy are likely due to practice/familiarity effects given that this improvement in MemTrax performance is also seen in the 23 participants without chemobrain after chemotherapy.

Another important point is that there is currently a major related issue in the field of Alzheimer’s disease (AD), recognition of subjective cognitive impairment might be an early indicator of mild cognitive impairment and early AD [30]. However, such complaints are relatively common, particularly among the elderly, and they may not be related to any underlying or significant pathology [31]. The data presented here suggest that situation with chemobrain may be similar to the field of AD, where self-report is not absolutely indicative of biological impairment.

### Limitations

1. This is a prospective study with a short duration, one week after the last chemotherapy treatment cycle and relatively small sample size of 41 participants.
2. Different chemotherapies are included in our study by design which covers a wider range of treatments which introduced more variables since they each could have unique effects on cognition.
3. Tests were administered both in person and remotely over phone, which may introduce quality control issues.

## Conclusion

In conclusion, we report for the first time that breast cancer patients in China experienced chemobrain after chemotherapy similar to their counterparts in other cultures and that the validated subjective assessment instrument FACT-Cog scale could detect the perceived impairments associated with chemobrain. However, the objective MemTrax memory test did not detect these impairments. Further studies with more patients and longer duration are needed to determine to what extent of the severity of chemobrain is related to the cognitive functions measured by MemTrax. If our results here are confirmed in a larger study, MemTrax may offer an opportunity for chemobrain patients to gain more insight into their condition and provide reassurance of their physical wellbeing for a speedy recovery in addition to means for continuous monitoring and caring for these patients by their medical providers.

### Supplementary Information

Supplementary Material 1.

## Data Availability

The data that support the findings of this study are not openly available due to reasons of sensitivity and are available from the corresponding author upon reasonable request.
