# Supplementary material for: Toward better understanding and management of chemobrain: the potential utilities of the MemTrax memory test
Source: BMC Womens Health. 2024 Jul 17;24:406. doi: 10.1186/s12905-024-03251-4 (PMC11253354; doi:10.1186/s12905-024-03251-4)
Supplement: Supplementary file 1 — Supplementary Material 1. [file 12905_2024_3251_MOESM1_ESM.docx]

**Clinical Characteristics Questionnaire**

Date：

CBM No.： Name： Tel NO.：

Date of Birth： / Education（years）： Nationality：

Height： CM Weight： KG BMI

Numbers of pregnancies

Total Chemotherapy Courses： Regimen：

Occupation： Nature of work： manual / mental

Living Condition： solitary (alone) / with family

Memory problems in the last two years: Yes（recent/distant recollections）/ No / Unknown

Memory is worse than it was five years ago？Yes/ No / Unknown

History of Cerebrovascular Diseases？Yes（Medication/Administration time： ） / No / Unknown

History of Diabetes？Yes（Medication/Administration time： ） / No / Unknown

History of Hypertension？Yes（Medication/Administration time： ） / No / Unknown

History of Coronary Heart Disease？Yes / No / Unknown

History of Hyperlipidemia？Yes / No / Unknown

History of Anemia？Yes / No / Unknown

History of Thyroid Disfunction？ hypothyroidism / hyperthyroidism / normal / unknown

History of Brain Trauma？ Yes / No

History of Epilepsy？ Yes / No

History of Carbon-Monoxide poisoning？ Yes / No

History of Operation under general anesthesia？ Yes / No

History of Alzheimer's Disease？ Yes / No / Unknown

History of other disease？

History of Psychiatric Illnesses？

Sleeping time per day: Hours Napping time？ Hour/No

Smoking？ Yes（ cigarettes/ day） / No

Alcohol？ Yes（ grams/day） / No

Drinking green tea？yes（ ML/day） / No

Left-handed/ Right-handed

Physical Exercises：methods： times of per week： length： minutes；

Hobbies：mahjong, playing cards, square dancing, Tai Chi, watching TV, etc.

Fatigue： No / affecting work and life（Yes/No）

Do you have the faith to overcome the disease？ Yes/No

Frequency of Sexual life： less than 4 times/month、4-8times/month、more than 8 times/month

Are you satisfied with your sexual life？ satisfied /not too bad /dissatisfied
